# Supplementary material for: Distinct Bioenergetic Features of Human Invariant Natural Killer T Cells Enable Retained Functions in Nutrient-Deprived States
Source: Front Immunol. 2021 Aug 9;12:700374. doi: 10.3389/fimmu.2021.700374 (PMC8380770; doi:10.3389/fimmu.2021.700374)
Supplement: Supplementary file 1 [file DataSheet_1.pdf]

Supplementary Material

Khurana et al. (2021)

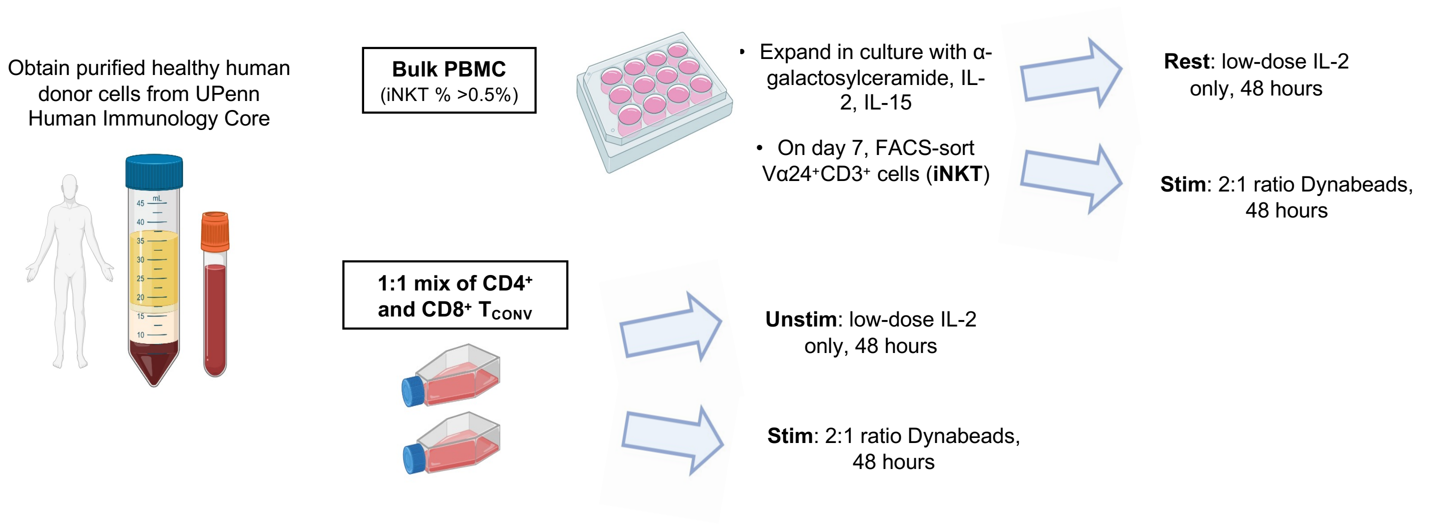

Supplementary Figure 1: Expansion and stimulation scheme for purifying healthy human donor PBMC-derived iNKT cells and conventional T cells (T_{CONV}). Schematic depicting obtaining and expanding purified human donor cells from UPenn Human Immunology Core. For all studies, iNKT cells were expanded, FACS-sorted, and rested as indicated. $CD4^+$ and $CD8^+$ conventional T cells (T_{CONV}) from matched sets of human donors were mixed at a 1:1 ratio to ensure equal composition of these subsets for consistency in all studies. Matched iNKT cells T_{CONV} and were subjected to 48 hours of "rest" and "stimulation" with CD3/CD28 Dynabeads as indicated. Images created using BioRender.

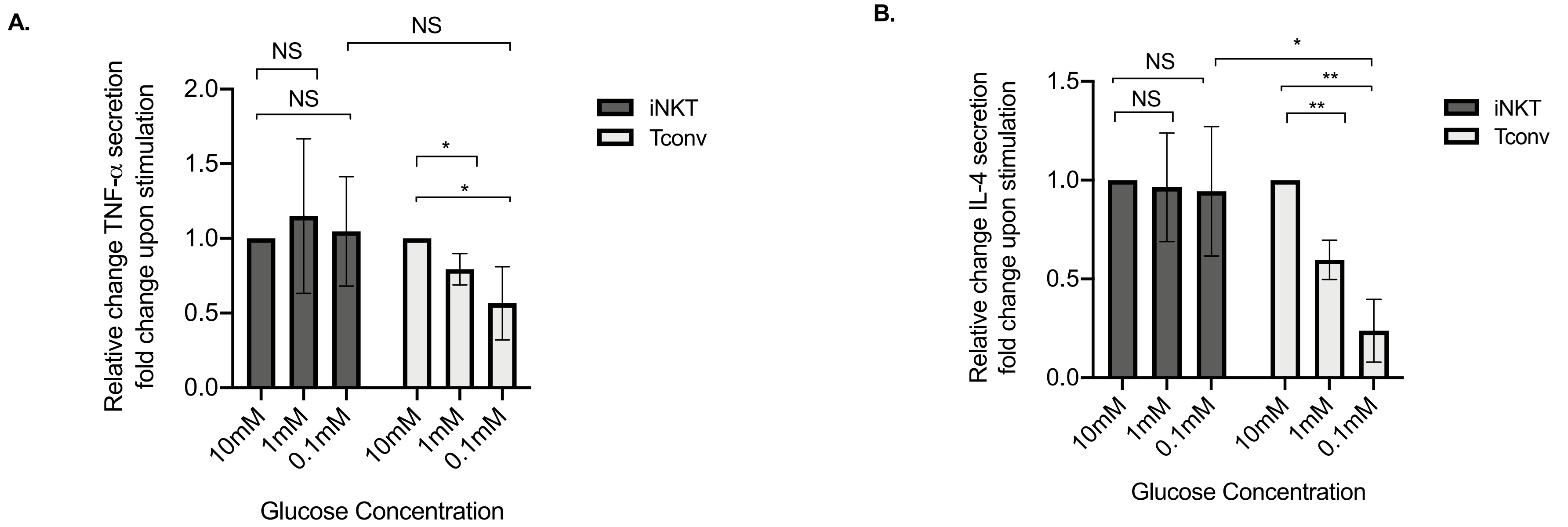

<u>Supplementary Figure 2</u>: Human iNKT cells maintain production of additional cytokines in glucose-depleted media relative to T_{CONV}. Supernatants were collected from rested and stimulated iNKT cells and T_{CONV} cultured in 10mM, 1mM, or 0.1mM glucose after 48 hours. Secreted TNF-α **(A)** and IL-4 **(B)** were quantified via MSD Multiplex Assay. Summary data of percent change in fold change upon stimulation for 3 independent, matched human donors depicted in bar graphs for stimulated iNKT cells (dark grey) and stimulated T_{CONV} (light grey). Each biological replicate was collected, stimulated, and harvested independently, and supernatant samples were run on the same MSD plate together. Asterisks indicate statistical significance (* $p<0.05$, ** $p<0.01$, *** $p<0.001$) determined by unpaired, two-way student's *t* tests.

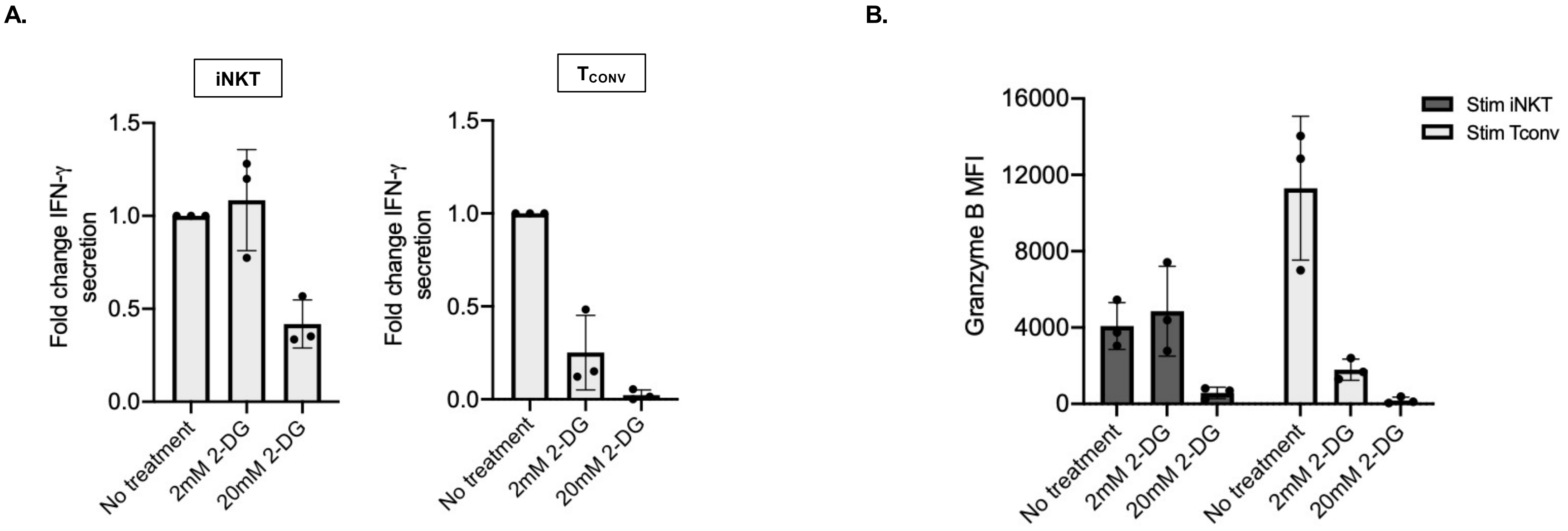

<u>Supplementary Figure 3</u>: Human iNKT cells are less sensitive to pharmacological inhibition of glycolysis than T_{CONV}. (A) Sorted PBMC-derived iNKT cells and T_{CONV} were rested or stimulated for 48 hours in complete AIM V media with low-dose IL-2 (30U/mL) only or containing either 2mM or 20mM 2-Deoxy-D-glucose (2-DG). Supernatants from rested and stimulated iNKT cells and T_{CONV} cultured in each condition were collected after 48 hours and profiled for levels of IFN-γ via ELISA. Fold change in IFN-γ secretion upon stimulation is displayed for iNKT cells (left) and T_{CONV} (right) relative to rest and unstimulated conditions, respectively. **(B)** Stimulated iNKT cells and T_{CONV} were stained for intracellular Granzyme B or isotype control after 48 hours of indicated treatments. Bar graph depicts mean fluorescence intensity (MFI) of granzyme B relative to isotype control. Each dot represents independent, matched human donor sample run in independent experiments.

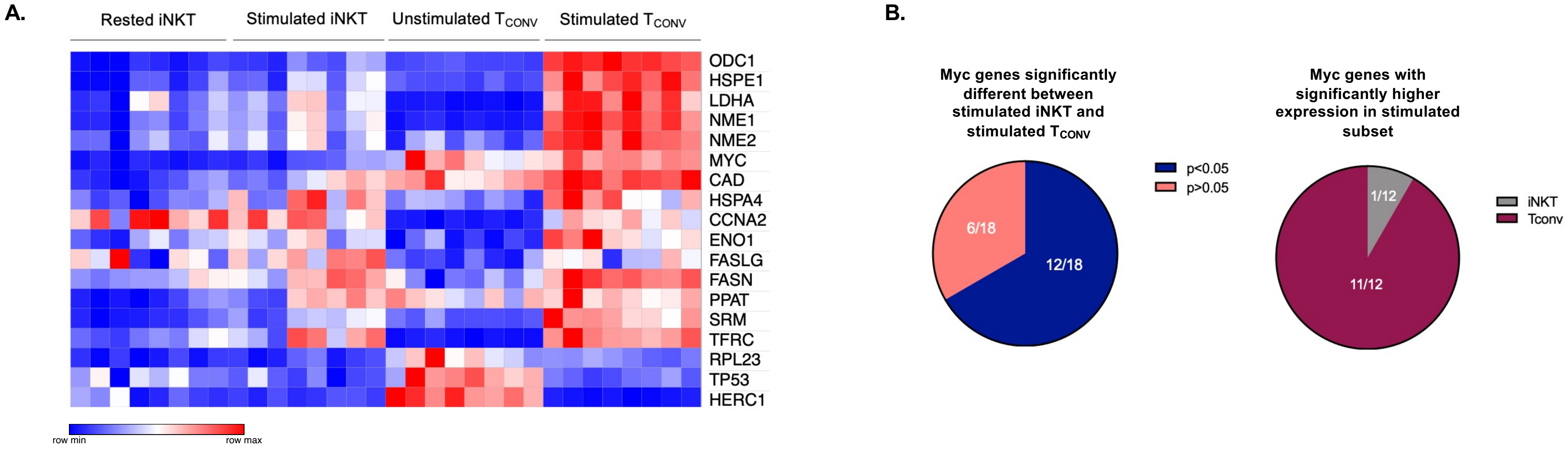

<u>Supplementary Figure 4</u>: Stimulated human iNKT cells have lower expression of Myc signaling genes than T_{CONV}. (A) Heatmap of n=8 independent, matched healthy human donor-derived rested and stimulated iNKT and T_{CONV} relative expression of Myc pathway genes in NanoString nCounter Human Metabolic Pathways probe set. Genes with counts under 100 were eliminated from analysis. Coloring indicates relative expression of each gene, from low (blue) to high (red). Heatmap generated on Morpheus. **(B)** Pie charts displaying statistical analysis of NanoString Myc pathway genes from (A). Proportions of genes significantly different in stimulated iNKT vs. stimulated T_{CONV} depicted (left), and of those genes, percentages significantly higher in T_{CONV} and iNKT shown (right). statistical significance was determined by unpaired, two-way student's *t* tests. Each set of matched donor biological replicates was independently collected, stimulated, and harvested for mRNA and all samples were run and analyzed on NanoString together.

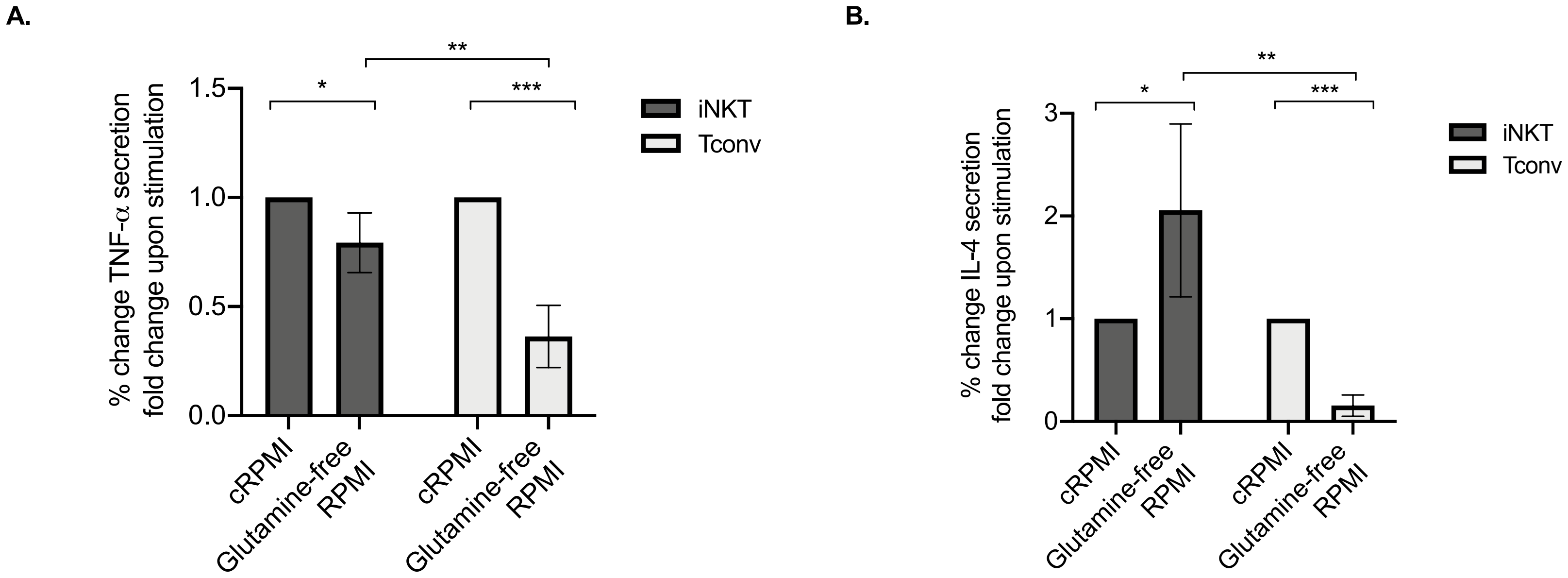

Supplementary Figure 5: Glutamine is not required for secretion of additional cytokines in human iNKT cells upon stimulation. Supernatants were collected from rested and stimulated iNKT cells and T_{CONV} cultured in either complete RPMI media or glutamine-depleted RPMI media conditions after 48 hours. Secreted TNF-α **(A)** and IL-4 **(B)** were quantified via MSD Multiplex Assay. Summary data of percent change in fold change upon stimulation for 4 independent, matched human donors depicted in bar graphs for stimulated iNKT cells (dark grey) and stimulated T_{CONV} (light grey). Each biological replicate was collected, stimulated, and harvested independently, and supernatant samples were run on the same MSD plate together. Asterisks indicate statistical significance (* $p<0.05$, ** $p<0.01$, *** $p<0.001$) determined by unpaired, two-way student's *t* tests.

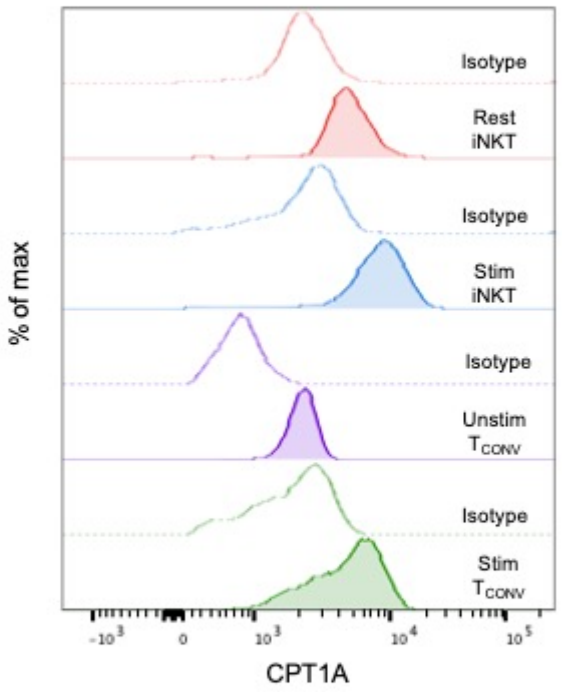

<u>Supplementary Figure 6</u>: Intracellular Cpt1a expression in rested and stimulated cell subsets. iNKT cells and T_{CONV} were rested or stimulated for 48 hours as indicated in Supplemental Figure 1. Cells were harvested and stained for intracellular flow cytometry expression of Cpt1a or isotype control. Representative histograms depicted for each cell type; isotypes (no shading) and Cpt1a graphs (shaded) indicated for each cell type, colored by population. Data representative of n=4 matched human donor biological replicates run in independent experiments.

Supplementary Table 1. Myc pathway gene set expression in stimulated iNKT vs. T_{CONV}

Gene name	p-value	q-value	Higher expressing cell subset
ODC1	1.7854E-10	1.4426E-09	T_{CONV}
MYC	1.9047E-08	7.6950E-08	T_{CONV}
HSPE1	1.5100E-06	4.0670E-06	T_{CONV}
NME1	3.0130E-06	6.0862E-06	T_{CONV}
NME2	4.3946E-06	7.1017E-06	T_{CONV}
CAD	1.5126E-05	2.0369E-05	T_{CONV}
HERC1	6.1085E-05	7.0510E-05	iNKT
LDHA	1.2875E-04	1.3004E-04	T_{CONV}
SRM	2.3461E-04	2.1063E-04	T_{CONV}
RPL23	1.0744E-03	8.6815E-04	T_{CONV}
FASN	1.5111E-02	1.1100E-02	T_{CONV}
ENO1	1.8666E-02	1.2568E-02	T_{CONV}
TFRC	5.8868E-02	3.6589E-02	T_{CONV}
FASLG	7.2688E-02	4.1952E-02	iNKT
PPAT	1.3568E-01	7.3088E-02	T_{CONV}
CCNA2	1.8492E-01	9.3383E-02	iNKT
HSPA4	4.1727E-01	1.9833E-01	T_{CONV}
TP53	7.4413E-01	3.3403E-01	T_{CONV}

Supplementary Table 2. Fatty acid oxidation (FAO) pathway gene set expression in stimulated iNKT vs. T_{CONV}

Gene name	p-value	q-value	Higher expressing cell subset
CPT1A	1.8304E-08	9.2435E-08	iNKT
ACAA2	2.2474E-07	5.6746E-07	iNKT
ACAT2	1.7960E-05	3.0232E-05	iNKT
ACOX1	1.1788E-03	1.4883E-03	iNKT
ACAT1	8.2236E-03	8.3058E-03	iNKT
PRKAB2	1.9726E-01	1.6603E-01	iNKT
HADH	2.3673E-01	1.6985E-01	iNKT
PRKAG2	2.6907E-01	1.6985E-01	T_{CONV}
ECHS1	9.4583E-01	5.3072E-01	iNKT
